# Supplementary material for: Spatial Variation in Stream-Water Bacterial Communities Across Sites Differing in Wild Chinese Giant Salamander Detection Frequency
Source: Biology (Basel). 2026 Sep 18;15(18):1647. doi: 10.3390/biology15181647 (PMC13604818; doi:10.3390/biology15181647)
Supplement: Supplementary file 1 [file biology-15-01647-s001.zip › Data Related to “Spatial Variation in Stream-Water Bacterial Communities across Sites Differing in Wild Chinese Giant Salamander Detection Frequency”/Microbial Data/result/03.TaxonVisual/heatmap/sample/heatmap.sample.p.cluster.pdf]

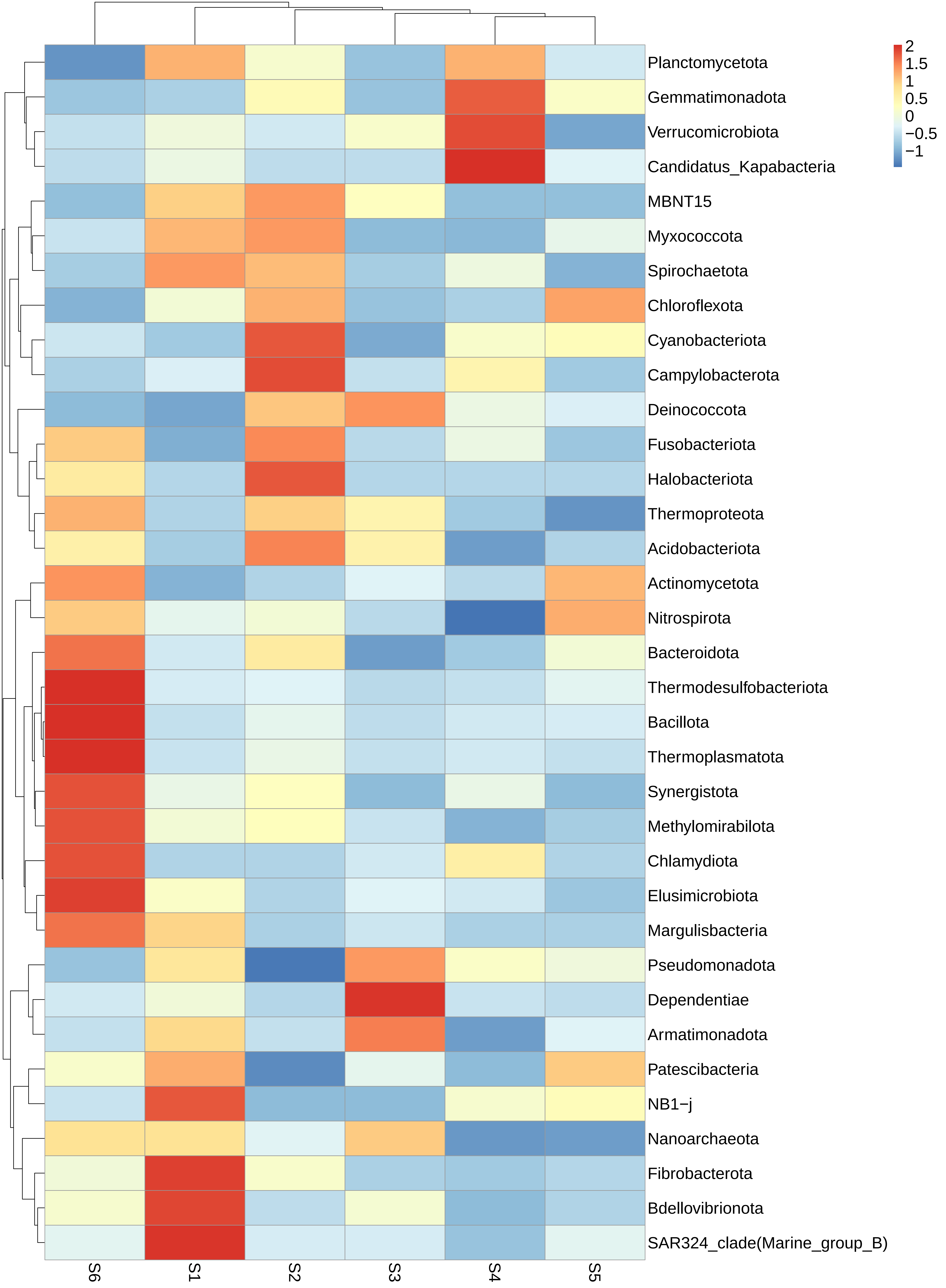
Planctomycetota
Gemmatimonadota
Verrucomicrobiota
Candidatus_Kapabacteria
MBNT15
Myxococcota
Spirochaetota
Chloroflexota
Cyanobacteriota
Campylobacterota
Deinococcota
Fusobacteriota
Halobacteriota
Thermoproteota
Acidobacteriota
Actinomycetota
Nitrospirota
Bacteroidota
Thermodesulfobacteriota
Bacillota
Thermoplasmatota
Synergistota
Methylomirabilota
Chlamydiota
Elusimicrobiota
Margulisbacteria
Pseudomonadota
Dependentiae
Armatimonadota
Patescibacteria
NB1−j
Nanoarchaeota
Fibrobacterota
Bdellovibrionota
SAR324_clade(Marine_group_B)
S6
S1
S2
S3
S4
S5
2
1.5
1
0.5
0
−0.5
−1
